# Spatial and seasonal patterns of FMD primary outbreaks in cattle in Zimbabwe between 1931 and 2016

**DOI:** 10.1186/s13567-019-0690-7

**Published:** 2019-09-24

**Authors:** Laure Guerrini, Davies Mubika Pfukenyi, Eric Etter, Jérémy Bouyer, Chenjerai Njagu, Felistas Ndhlovu, Mathieu Bourgarel, Michel de Garine-Wichatitsky, Chris Foggin, Vladimir Grosbois, Alexandre Caron

**Affiliations:** 1 ASTRE, CIRAD, INRA, Université de Montpellier, Montpellier, France; 2 CIRAD, RP-PCP, UMR ASTRE, Harare, Zimbabwe; 3 Department of Clinical Veterinary Studies, Faculty of Veterinary Science, University of Zimbabwe, Harare, Zimbabwe; 4 Epidemiology Section, Department of Production Animals Studies, Faculty of Veterinary Science, University of Pretoria, Pretoria, South Africa; 5 Governmental Veterinary Services, Harare, Zimbabwe; 6 Faculty of Veterinary Medicine, Kasetsart University, Bangkok, Thailand; 7 Victoria Falls Wildlife Trust, P O Box 159, Victoria Falls, Zimbabwe; 8 Faculdade de Veterinaria, Universidade Eduardo Mondlane, Maputo, Mozambique

## Abstract

Foot and mouth disease (FMD) is an important livestock disease impacting mainly intensive production systems. In southern Africa, the FMD virus is maintained in wildlife and its control is therefore complicated. However, FMD control is an important task to allow countries access to lucrative foreign meat market and veterinary services implement drastic control measures on livestock populations living in the periphery of protected areas, negatively impacting local small-scale livestock producers. This study investigated FMD primary outbreak data in Zimbabwe from 1931 to 2016 to describe the spatio-temporal distribution of FMD outbreaks and their potential drivers. The results suggest that: (i) FMD outbreaks were not randomly distributed in space across Zimbabwe but are clustered in the Southeast Lowveld (SEL); (ii) the proximity of protected areas with African buffalos was potentially responsible for primary FMD outbreaks in cattle; (iii) rainfall per se was not associated with FMD outbreaks, but seasons impacted the temporal occurrence of FMD outbreaks across regions; (iv) the frequency of FMD outbreaks increased during periods of major socio-economic and political crisis. The differences between the spatial clusters and other areas in Zimbabwe presenting similar buffalo/cattle interfaces but with fewer FMD outbreaks can be interpreted in light of the recent better understanding of wildlife/livestock interactions in these areas. The types of wildlife/livestock interfaces are hypothesized to be the key drivers of contacts between wildlife and livestock, triggering a risk of FMD inter-species spillover. The management of wildlife/livestock interfaces is therefore crucial for the control of FMD in southern Africa.

## Introduction

Foot and Mouth Disease (FMD), known since the sixteenth century [1], is a highly contagious viral disease (single-stranded RNA virus), infecting domestic and wild cloven-hoofed animals [2]. The mortality due to FMD is relatively low while its morbidity can be low to high depending on the circulating strain, including sometimes significant production losses. Once FMD is introduced in an animal production system, the virus spread easily, potentially impacting production outputs. Listed as a notifiable disease by the World Organization for Animal Health (OIE), FMD is therefore an important transboundary animal disease with consequences for international trade. With a few exceptions, FMD outbreaks have historically been observed in most areas of the world where significant livestock productions occur [1].

FMD has been the focus of intensive research, surveillance and control programs culminating in its eradication from Europe in the 20th century [3]. Today, the disease is still circulating in Asia, the Middle-East and Africa [4] with infrequent re-introduction in other areas (e.g. The United Kingdom in 2001) where it triggers devastating economic consequences [5]. After the successful rinderpest eradication campaign, the United Nations organization for food and agriculture (FAO) and OIE put in place the Progressive Control Pathway (PCP) to assist endemic countries in the control of FMD [6, 7].

In Africa, numerous serotypes of FMD, including the three South African Territories (SAT) serotypes, are heterogeneously distributed [8, 9]. The epidemiological picture is complex as SAT FMD viruses can be maintained in wildlife species, in particular the African buffalo (*Syncerus caffer caffer*), a confirmed maintenance host [10] and some of their life-history traits seem to differ from other strains (slower spread, more asymptomatic, environmental persistence) [11, 12]. The presence of numerous and large protected areas in Southern and Eastern Africa, with important wildlife populations creates extensive wildlife/livestock interfaces and therefore, complicates the control of FMD [13]. The surveillance and control of FMD in southern Africa differs from the other African regions [14]. Southern African countries have always considered the control of FMD as one of the main priorities of veterinary services since the colonial era. During this period, it has even been suggested that the disease was used to control people movements and livelihoods [15, 16]. The main objective of FMD control for the southern African beef trade was to access more lucrative markets (most recently the European markets). The strategies to control FMD were (and still are largely) based on zonation where free-of-disease zones are separated from infected zones (centered on protected areas hosting infected buffalo populations) by protection zones dedicated to vaccination and surveillance. In addition in southern Africa, the control of FMD is particularly complex because the epidemiology of the disease is associated with important conservation and development issues [17–19].

Recent studies have targeted different aspects of FMD epidemiology in Africa: investigation on the role of wildlife/livestock interfaces on FMD inter-species transmission [12, 20, 21], the ecology of the different serotypes [22–24], the risk factors linked to animal husbandry [25] and the role of the environment [26]. Taking into account this new knowledge on the disease, sound risk-based surveillance and control strategies for FMD are needed and should be more respectful of local livelihoods and the environment [27].

In Zimbabwe, FMD has been occurring at least since the end of the 18th century and the role of wildlife in spreading the virus has been suspected for a long time [28–30]. Beef trade with Europe in the 80’s and 90’s required intensive FMD control [31]. However, the deterioration in the socio-economic situation witnessed in Zimbabwe at the end of the last century, resulted in a drastic reduction of veterinary services’ ability to control the disease, and eventually, in an upsurge of FMD outbreaks. As a consequence, the control of FMD outbreaks was reduced to ring vaccination around infected cattle populations. FMD outbreaks are known to be located in specific geographic areas and to be driven by abiotic factors, but, so far, apart from early mapping by Condy [30], no analysis of the spatial and temporal distribution of outbreaks was implemented to better understand the dynamic patterns of this disease and its drivers.

In the present paper, a spatio-temporal analysis was implemented on FMD outbreak data from 1931 to 2016 to describe the spatial heterogeneity and the risk period(s) of FMD outbreaks. The proximity of protected (conservation) areas and the seasonality of FMD outbreaks, both factors that could contribute to FMD dynamics in Zimbabwe were also analyzed. This study should contribute to identify hotspots and drivers associated with FMD outbreaks, suggests mechanisms for disease emergence at the wildlife/livestock interface and is expected to provide useful information to decision makers for tailoring risk-based surveillance of FMD in Zimbabwe.

## Materials and methods

### Study design and data collection

Our study was performed at a national scale, in the 8 provinces of Zimbabwe.

**Figure 1:**
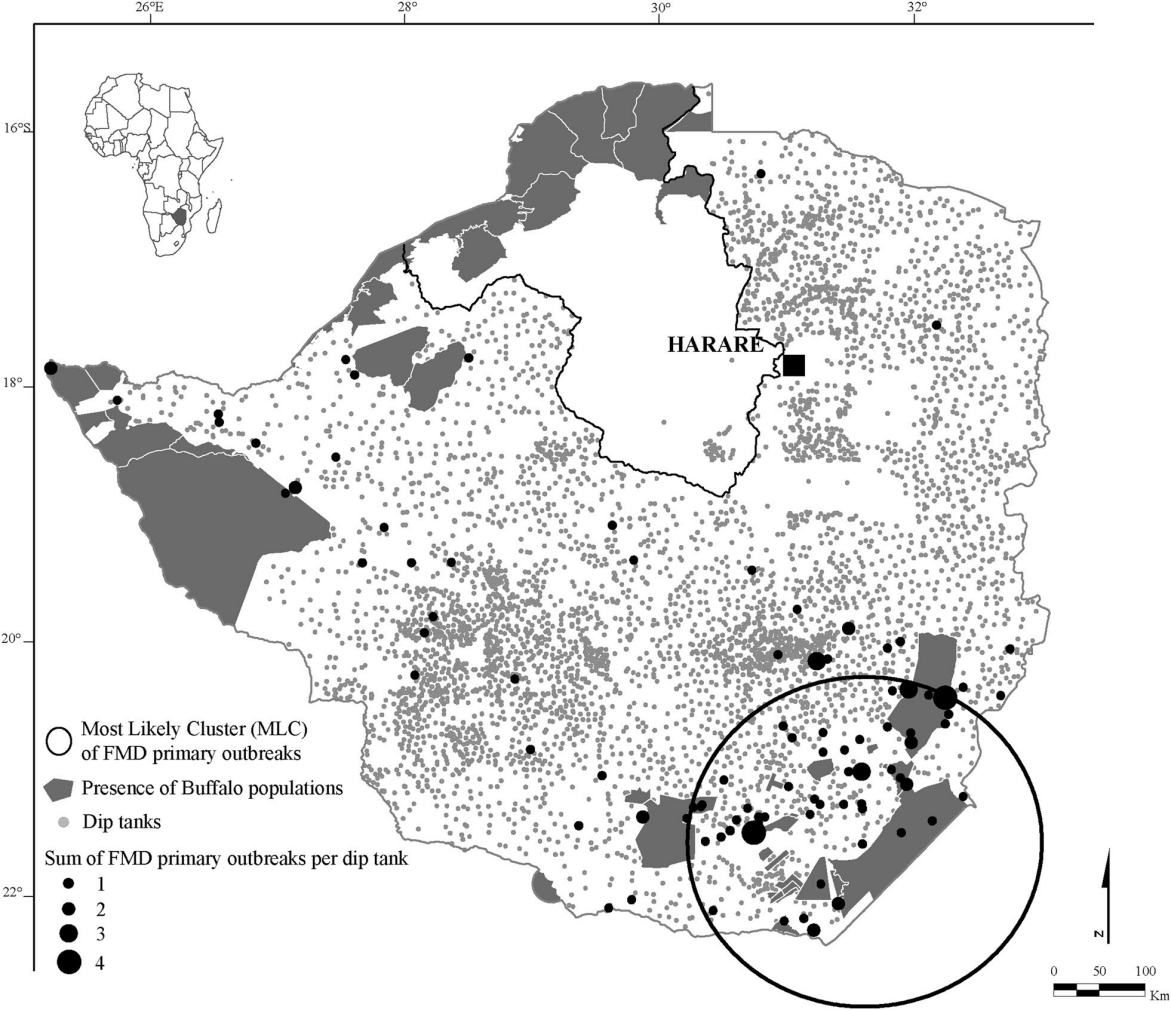
**Geographic location of dip tanks and foot and mouth disease primary outbreaks.** The dip tanks are presented in black and grey dot (*n* = 4960). The FMD primary outbreaks are presented as black dots (the size of the dots is proportional to the number of outbreaks, *n* = 110) from 1931 to 2016 in Zimbabwe. The protected areas (where African Buffalo populations are present) are presented as grey areas. The dot-line represented the Mashonaland West province for which the dataset for dip tanks location was not complete.

The official Zimbabwe FMD outbreaks database was obtained from the Department of Livestock and Veterinary Services of the Ministry of Agriculture, Mechanization and Irrigation Development—Zimbabwe (DLVS). We identified the primary outbreaks based on two criteria: (1) clusters of outbreaks were identified by their spatio-temporal distance (separated by time and/or locality) and within clusters, an outbreak was classified as primary if it was the first occurring within a detected cluster; and (2) we benefitted from the expertise of veterinary staff (including staff from the epidemiology and wildlife veterinary units of the governmental veterinary services) who either knew about or directly followed the occurrence of the recorded outbreaks. During the period 1931 to 2016, a total of 110 primary outbreaks were recorded. In general in southern Africa, serotypes A, O and C are only occurring in Tanzania with rare incursions of A and O most probably through importation of contaminated material (i.e. A in South Africa, A and O in Angola and South Africa, [32]). Only the SAT serotypes are known to be present in Zimbabwe and we assumed that all the primary outbreaks considered in this study were SAT outbreaks. For each event, the month and the geographical coordinates of the dip tank where the diagnosis was done were documented (Figure 1).

A monthly average rainfall was calculated for each province from 1931 to 1997 (the full monthly rainfall data was incomplete after 1997) using the rainfall time series from the Agroclimatic database management system [33].

### Spatial cluster detection

We tested the hypothesis of Condy [30] who observed some spatial cluster patterns of FMD primary outbreaks in Zimbabwe. In order to detect these clusters, a dataset from DVLS of all dip tank locations in the country (except dip tanks from the Mashonaland West province, dataset being updated at the time of the study) was used. This dataset provided the baseline data to compare infected location vs. non-infected location (both at the dip tank level). The distribution of dip tanks in Zimbabwe can also be used as a proxy of cattle distribution and densities as each dip tank has been built to serve a population of about 1500 heads of cattle.

In order to identify geographic clusters of FMD outbreaks in Zimbabwe, and to assess their statistical significance, spatial scan statistics available in the SaTScan™ software, version 9.4.2 (Kulldorff and Information Management Services, 2006) were used. Specifically, the so-called “Bernouilli model” to detect spatial clusters was used. This method accounts for the spatial distribution of all the diptanks in the country. Each diptank is characterized as being either a case (i.e. a diptank that has experienced at least one FMD outbreak over the study period) or a non-case (i.e. a diptank that has never experienced any FMD outbreak over the study period). The method allows the identification of circular areas within which the proportion of cases is larger than expected under the null hypothesis of a spatially homogeneous incidence rate. In this method a series of circles of varying radii is constructed around each case (i.e. each diptank that has experienced at least one outbreak). For each circle the alternative hypothesis is that there is an elevated risk of outbreak in the circle compared to that outside. A test statistics based on the numbers of cases and non-cases inside and outside the focal circle is computed (see [34] for a more detailed description of the test statistics used). Clusters were assessed up to scales at which 50% of all diptanks at which FMD has been detected are included in one cluster For each circle, a *p* value is computed using a permutation method in which the observed test statistics is compared to the distribution of the same test statistics obtained from data generated by randomly permuting the case and non-case status associated with each point (i.e. each disptank). Because in the permutation data sets the status of each point (i.e. each diptank) is randomly attributed, the distribution of the test statistics over permutation data sets provides a distribution of the test statistics under the null hypothesis of a spatially constant incidence rate. Clusters are considered as statistically significant whenever the observed value of the test statistics falls within the 5% largest values in the distribution of the test statistics obtained over the permutation data sets.

### Drivers of FMD outbreaks

Based on the literature, four potential drivers of FMD outbreaks were selected and their influence on FMD outbreaks tested: (i) the proximity of protected areas; (ii) seasons; (iii) water availability; (iv) political and economic contexts. Livestock practices, cropping calendars and climatic conditions are different across the country; therefore, geographic differences (i.e. differences among provinces or regions) in the influence of potential risk factors and in seasonal variation pattern were also assessed. Provinces were merged into three regions: the two provinces of Masvingo and Manicaland formed the Southeast Lowveld region (SEL); the two provinces of Matabeleland North and Matabeleland South formed the Matabeleland region; both regions are characterized by extensive wildlife/livestock interfaces. The rest of the four provinces formed the Central region.

### Distance to protected (conservation) areas on the variation in the probability of FMD outbreaks

Following the results of the cluster hypothesis, we hypothesized the maintenance role played by the African buffalo population in the FMD outbreak patterns in Zimbabwe as it has been shown previously in South Africa [9, 35]. Protected areas with known buffalo populations were selected and used as a proxy of FMD presence in the buffalo population [36] (Figure 1). The geographic coordinates of the dip tanks outside protected areas was considered (*n* = 4850 points with non-FMD outbreak and *n* = 110 points where FMD outbreaks occurred). The Euclidian distance from dip tanks to the nearest protected areas was calculated using the ArcGIS 10.2 software (ESRI; Redlands, USA). The influence of the distance to protected areas on the FMD outbreaks was studied using a generalized linear model on the 8 provinces of the country and on the SEL and Matabeleland regions.

### Temporal and seasonal variation analysis

Seasons determine environmental variables (e.g. rainfall, temperature) that can influence FMD epidemiology as well as agricultural calendar determining cropping and herding calendars. Seasons were defined as: rainy season (November to March), cold dry season (April to July) and hot dry season (August to October). For delimitations between years to match with the succession of seasons, the year was modified as to start in April, at the transition between the rainy season and the cold and dry season (and not in January in the middle of the rainy season). According to this delimitation, year Y started in April Y and ended in March Y+1.

Seasonal variation in the occurrence of FMD outbreaks was explored using Generalized Linear Models where the total number of outbreaks during the study period (i.e. from April 1931 (beginning of year 1931) to March 2016 (end of year 2015)) in a given region and during a given month was the Poisson distributed response variable. A third order polynomial function of a quantitative month variable (where April was attributed the value 1 and March the value 12) was included as an explanatory variable in order to depict the seasonal variation pattern. The region categorical variable and the interaction between region and the seasonal pattern terms were also included in the model in order to assess differences among regions in the outbreak incidence seasonal pattern. The statistical significance of the explanatory variables was tested using Likelihood Ratio Tests.

Seasonality of rainfall was graphically displayed by plotting for each province the mean of rainfall in each month over the years for which full rainfall data was available, i.e. from April 1931 (beginning of year 1931) to March 1997 (end of year 1996).

### Effect of water availability on inter-annual variation in FMD outbreaks

Water availability was considered as a potential risk factor because under dry conditions limited access to water is likely to result in increased contacts among cattle and between cattle and buffalo at the few remaining water points, which could in turn result in the intensification of FMD virus circulation within and between the cattle and the buffalo compartments. Data on water availability was not available but rainfall cumulated over 1 year at the end of the rainy season was considered as a proxy for the replenishment level of water reserves (water points, water courses, wells, etc.…) and considered as potentially influencing the incidence of primary FMD outbreaks over the next 12 months. The number of FMD outbreaks in each province in year Y (from April Y to March Y+1) was thus related to the sum of rainfall over the months of year Y−1 in the same province (from April Y−1 to March Y) in Generalized Linear Models where the number of outbreaks was the Poisson distributed response variable and rainfall during the preceding year, province and the interaction between province and rainfall in the preceding year were included as explanatory variables. The statistical significance of the explanatory variables was tested using Likelihood Ratio Tests.

### Variation in the number of FMD outbreaks among historical periods

Four periods were defined based on empirical evidences of the political and socio-economic history of Zimbabwe as well as expertise from Zimbabwean personal from DLVS and used to estimate the relative risk of FMD between them. The first period from 1931 to 1969 corresponded to the colonial era, economically stable even during the international sanctions, the veterinary services were assumed to be efficient in Southern Rhodesia compared to the following 1970 to 1979 period when the independence war of Zimbabwe considerably weakened the animal health surveillance system. The third period from 1980 to 2000 coincided with the emergence of the Zimbabwean state, with a brisk economic recovery, a regain of stability and a recovery of national extension services including the animal disease surveillance. The capacity to export beef to the European Union during this period proved this regain in surveillance capacity [37]. The fourth and last period from 2000 to 2016 corresponded to the economic crisis following the land reform, which impacted the means of the veterinary services and reduced the possibility of control and surveillance, leading to another collapse of the animal health surveillance system. This classification is fairly similar to the one used in a recent study [38]. Variation in the occurrence of FMD outbreaks at the national scale among these periods was tested using a Generalized Linear Model (GLM). In this model the response variable was the number of outbreaks recorded in a year and the explanatory variable was the period variable which included the categories defined above. As the response variable was a count variable, it was considered to follow a Poisson distribution. The statistical significance of the explanatory variable was tested using Likelihood Ratio Tests.

All analyses were performed using the R 3.3.1 software [39].

## Results

### Spatial clustering

The Kulldorff’s spatial scan statistic method produced four clusters during the detection step and retained only one after the inference step. The MLC contained 548 dip tanks, mainly located in the Masvingo Province in the South-East of Zimbabwe presented a high risk area with the number of observed outbreaks greater than the adjusted expected number of outbreaks (LLR = 85.43, *p* < 10^−3^). The three non-significant secondary clusters contained each less than 7 dip tanks and were thus considered of little epidemiological relevance (Table 1).

**Table 1:** **Description of FMD clusters from the spatial analysis, 1931–2016**

Dip tanks (number)	Type^a^	Radius (km)^b^	Location^c^	Observed cases	Expected cases	RR^d^	LLR^e^	*p*-value
548	M	142.78	21.6 S, 31.6 E	68	5.95	13.95	85.43	10^−16^
6	S	0	19.95 S, 31.5 E	3	0.04	69.55	9.75	0.1
4	S	59.68	17.9 S, 25.3 E	3	0.09	34.76	7.7	0.5
2	S	14.9	18 S, 27.6 E	2	0.04	45.94	5.7	1

^a^M most likely cluster, S secondary cluster.

^b^Radius, distance between the center of cluster and his borders.

^c^Geographic coordinates of the center of the cluster.

^d^RR Relative risk inside the cluster, compared to the rest of the study area.

^e^LLR Log likelihood ratio.

### Proximity to protected (conservation) areas and FMD risk

**Figure 2:**
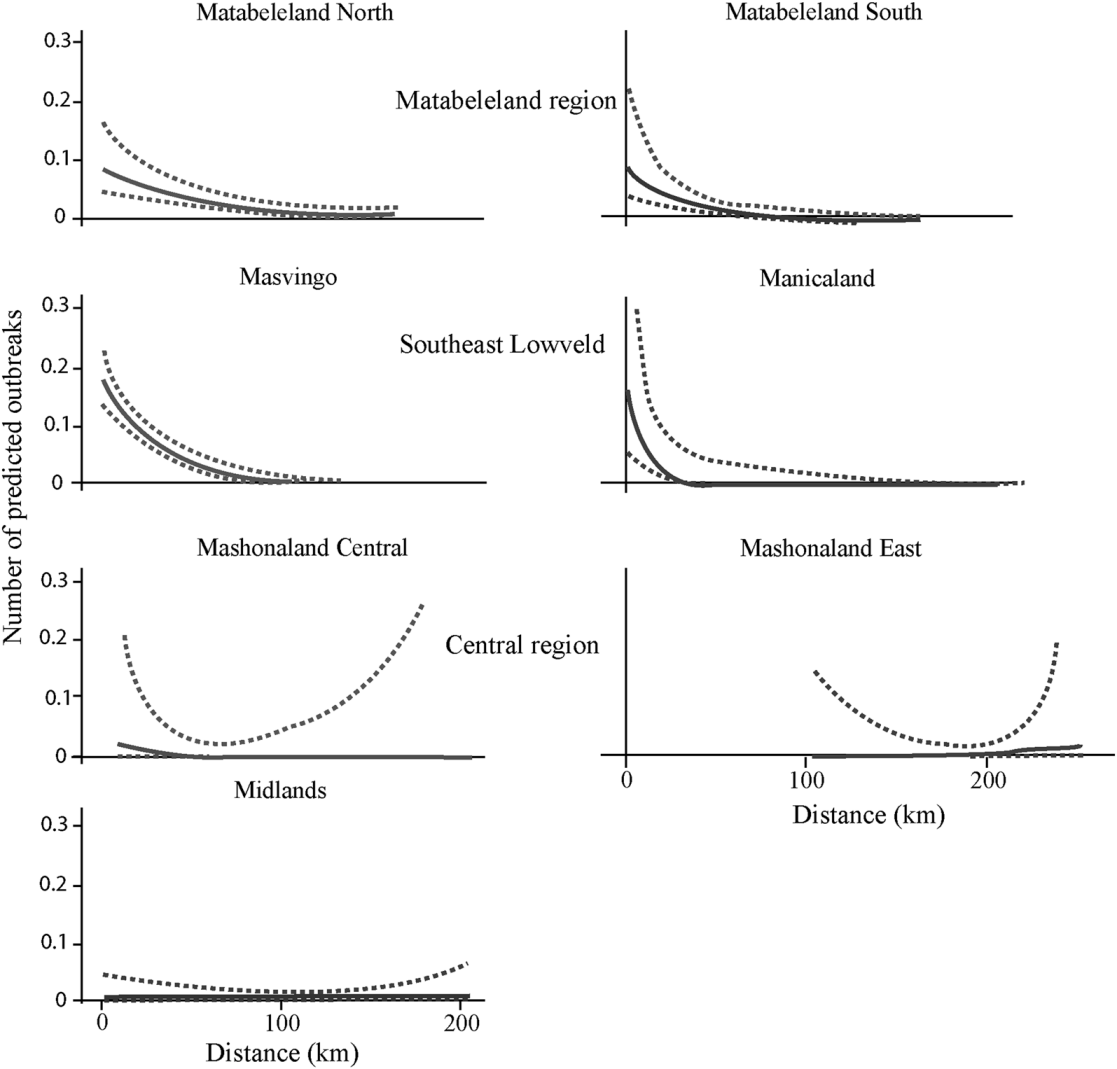
**FMD outbreaks predicted by the model related to the distance to protected areas.** The number of foot and mouth disease outbreaks in relation to the distance (in km) to the protected areas are predicted by the generalized linear model for the seven provinces in Zimbabwe.

The number of FMD outbreaks recorded over the study period at the dip tank level was significantly related with the distance to protected areas (Figure 2). This relationship differed among provinces (*p*-value for the interaction between province and distance to protected area: 0.002). The number of outbreaks clearly increased with proximity to protected areas in the provinces of the SEL and Matabeleland regions (Matabeleland North and South provinces) which include large protected areas and where most FMD outbreaks occurred. By contrast, in the provinces of the Central region which are far away from large protected areas containing African buffalo and where few FMD outbreaks occurred, no relationship was detected.

### Seasonal variation in the number of FMD outbreaks

**Figure 3:**
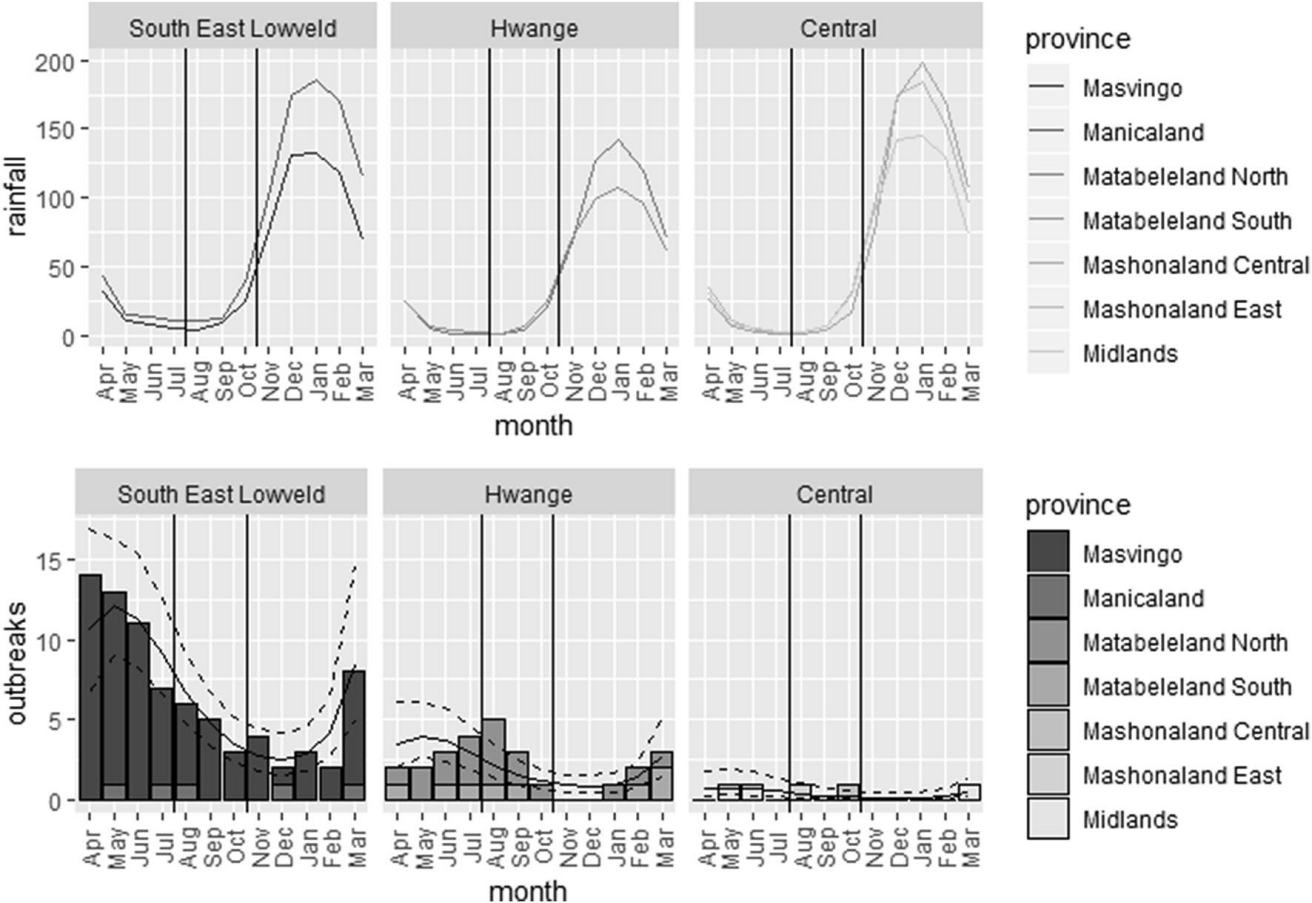
**Seasonal variation in the number of FMD outbreaks.** Top panel: mean of rainfall per month from 1931 to 2016, per season (rainy—November–March, cold-dry—April–July and hot-dry—August–October; separated by vertical lines) and per region (the South-East Lowveld included the Manicaland and Masvingo provinces, the Matabeleland region included the Matabeleland South and the Matabeleland North, the Central region included the Mashonaland Central, the Mashonaland West, the Mashonaland East and the Midlands provinces). Bottom panel: sum of foot and mouth disease outbreaks (bars) per month in the three regions of Zimbabwe as described above and predictions with 95% confidence interval (lines) of the statistical model selected to depict seasonal variation in the number of FMD outbreaks.

The interaction between region and the third order polynomial function of month was not statistically significant (Table 2; *p*-value = 0.23) suggesting either that the seasonal pattern of variation in the number of FMD primary outbreaks was similar in the three regions or that statistical power was too low to detect differences among regions. The main effects of region and of the third order polynomial function of month were highly significant (Table 2; *p*-value < 0.0001). The overall number of outbreaks was highest in the South East Loweld region, intermediate in the Hwange region and lowest in the central region. According to the third order polynomial function of month fitted to the data, FMD primary outbreak incidence was high from the end of the rainy season and all through the cold and dry season and low from the middle of the hot and dry season through most of the rainy season (Figure 3). However, it is clear that this pattern fits much better the outbreak records from the South East Loweld region than the outbreak records from the Hwange or the Central regions (Figure 3). For these two last regions, the number of primary outbreaks recorded is insufficient to provide a robust depiction of seasonal incidence patterns.

**Table 2:** **Seasonal variation in FMD primary outbreak incidence**

	LRT	df	*p*-value
Season (3^rd^ order polynomial of month)	30.29	3	< 0.0001
Region	21.6	2	< 0.0001
Season: region	8.08	6	0.23

The statistical significance of the main effects of season and month were tested in a model that did not contain any interaction between Region and season.

### Inter-annual variation in the occurrence of FMD outbreaks in relation with water availability

The number of outbreaks over the 12 month period from the beginning of the cold dry season to the end of the next rainy season was not statistically related to the cumulated rainfall over the previous 12 month period (Table 3, *p*-value = 0.16) even when possible heterogeneity among provinces in the influence of rainfall over the preceding year was taken into account (Table 3; *p*-value for the interaction between province and rainfall: 0.24). This suggests either that water availability does not influence the incidence of FMD outbreaks or that cumulated rainfall over 12 months at the end of the rainy season is not a good proxy for water availability over the next 12 months.

**Table 3:** **Rainfall patterns between years and FMD outbreaks**

	LRT	df	*p*-value
Rainfall over the preceding year	1.98	1	0.16
Province	138.96	6	< 0.0001
Rainfall over the preceding year: province	8.01	6	0.24

Statistical model for relationship over the period 1932–1996 between annual number of outbreaks at the province scale and rainfall over the preceding year.

### Variation in the number of FMD outbreaks among historical periods

**Figure 4:**
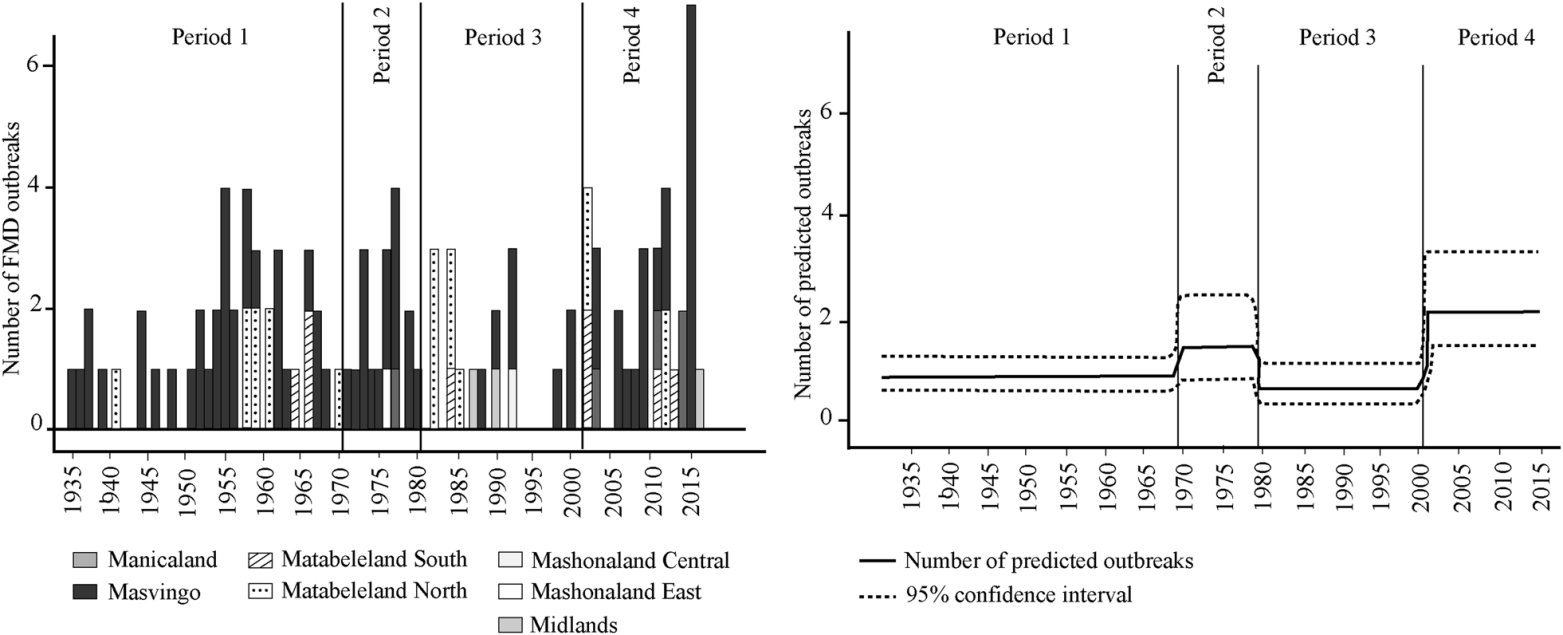
**Variation in the number of FMD outbreaks among historical periods.** Left panel: number of foot and mouth disease outbreaks from 1931 to 2016 in the seven provinces of Zimbabwe, per period (vertical black lines). Right panel: number of foot and mouth disease outbreaks predicted by the generalized linear model (continuous black line) and their 95% confidence interval (dotted lines).

The number of annual outbreaks at the national level varied significantly among historical period (*p*-value = 0.0036), Table 4. It was particularly high during two periods: the 1970–1979 and the 2000–2016 periods. The predictions of the GLM model for the four considered period presented in Figure 4 indicates similar trends. Interestingly, Period 3 and 4 are the only periods with outbreaks outside the SEL and Matabeleland regions.

**Table 4:** **FMD outbreaks among historical periods**

Parameters	LRT	df	*p*-value
Period	13.53	3	0.00362
1931–1969			0.28
1970–1979			0.03
1980–2000			Ref.
2000–2015			0.001

Statistical model for variation among historical periods in the annual number of primary outbreaks recorded at the national scale in Zimbabwe. The 1980–2000 period characterized by the prosperity following independence is considered as the reference period for pairwise comparisons among periods. The *p*-values associated with comparisons of each period with the reference period are obtained though.

## Discussion

The present study on FMD primary outbreaks between 1931 and 2016 provides insights on the spatial and seasonal patterns of the disease in Zimbabwe. The results of this study indicated that in Zimbabwe: (i) FMD outbreaks were not randomly distributed in space and time across the country as previously suggested 40 years ago by Condy [30] with the SEL being more prone to FMD outbreaks followed by the Matabeleland region and the Central regions; (ii) distance to protected areas was significantly associated with FMD primary outbreaks in the SEL and Matabeleland regions with the presence in both regions of extensive wildlife/livestock interfaces; (iii) seasonality but not rainfall influenced the occurrence of FMD outbreaks in both the SEL and Matabeleland regions but differently indicating a differential role of wildlife/livestock interfaces; (iv) and the political context and its socio-economic consequences influenced the occurrence of FMD outbreaks with political and economic instability being linked with a surge in primary outbreak numbers.

Some potential biases of the approach need to be considered. First, the dataset of primary outbreaks recorded over a period of 85 years was identified “manually” by local DLVS experts who closely monitored outbreaks in the last decades. The lack of availability of strain specificity (determined at the molecular level) for the majority of these outbreaks prevents the possibility to confirm that each of these data points is a unique primary outbreak or a combination of simultaneous outbreaks. However, each of our primary outbreaks is the starting point of a clear spatial and temporal series of secondary outbreaks that could be linked to one or more strains. Second, our primary outbreaks dataset represents a set of detected outbreaks and maybe not the complete picture of FMD outbreaks in Zimbabwe during the period covered. “Silent” FMD outbreaks have been suspected in cattle in southern Africa [24, 30]. This silent circulation of FMD in cattle in southern Africa is of concern and could be linked to undetected endemic situations in some cattle populations, including Zimbabwe. This study does not cover by design this invisible FMD circulation and only deals with those outbreaks that have been detected by DLVS in the covered period. Fourth, the political periods cannot be selected according to a pure quantitative method. They are characterized/delineated by important national socio-economic and political events or periods of the country and mostly based on expert opinion (but similar period have already been used for southern Africa) [38]). Finally, the African buffalo distribution reflects the current state of the population and could not track changes in buffalo populations in different ranches/protected areas (e.g. buffalo translocations in Zimbabwe for conservation purposes that were detected by a recent genetic study [40]). However, the control (i.e. shooting) of buffalo movements outside of protected areas by DLVS during most of the study period gave us confidence that this bias is limited [41].

The study identified a main cluster of primary outbreaks located in the SEL of Zimbabwe. The SEL is a dry area (average rainfall < 600 mm per year, [42]) identified by the Zimbabwean government as a low production zone for agricultural activities, suitable mainly for animal production including wildlife activities. Gonarezhou National Park and several conservancies (i.e. private protected areas) in the SEL host buffalo populations surrounded by communal land where small-scale farmers raise livestock (Figure 1). This region is therefore prone to wildlife/livestock interfaces where direct and indirect contacts between wild and domestic ruminants can promote disease transmission and in particular FMD spillover between cattle and buffalo [12, 20, 35]. The risk of pathogen and potentially disease spillover at wildlife/livestock in the SEL has already been demonstrated in Gonarezhou National Park [41, 43]. In addition, it is recognized that this region is prone to FMD outbreaks at buffalo/cattle interfaces [38, 44].

However, the SEL is not the only region in Zimbabwe with extensive wildlife/livestock interfaces. The largest national park in Zimbabwe, Hwange, hosts a large buffalo population. This area is classified by the Zimbabwean government also as a semi-arid zone, hosts significant livestock populations (Figure 1) and is also recognized as part of a regional cluster of FMD strains [45] and wildlife/livestock interactions do occur with potential for disease transmission as well [12, 46, 47]. Therefore, what makes the SEL more prone to FMD outbreaks compared to other similar zones such as the Matabeleland region?

First, there is no indication of a differential capacity of current and past district veterinary services to detect FMD outbreaks across regions [48]. Second, differences in cattle populations and movements between areas could explain the differences observed. Cattle densities between the two regions are comparable and insufficient information exists on informal transboundary cattle circulation [38, 49] to explain regional differences. Finally, to our knowledge, there are no obvious differences in cultural practices that can explain the patterns of FMD outbreaks observed [48]. However, the type of wildlife/livestock interfaces can have an impact on wild and domestic ungulate contacts and therefore pathogen transmission could contribute to the regional differences observed (Figure 2). At those interfaces, the distribution of key resources such as water and grazing drives wild and domestic ungulate distribution and dynamics.

Wildlife including buffalos and domestic ruminants depend nearly exclusively on natural water sources for drinking in the SEL region [50] and to lesser extent in the Hwange region. Water availability (e.g. waterholes, river pools) decreases as the dry season progresses to reach its lowest level during the hot and dry season [51, 52]. At that time of year cattle and buffalo rely exclusively on remaining water sources, where they can be in direct and indirect contacts potentially resulting in pathogen spillove [12]. Empirical [53] and modelling [52] evidence of increased contact frequency within and between cattle and wildlife populations in situations of low water and forage resources availability in the SEL region have already been reported. For example, in Gonarezhou, the interface between the park and Malipati village is the Mwenezi River that retains only a handful of water pools during the dry seasons, attracting both wildlife and cattle [50]. Under the hypothesis that scarce water resources generate favorable situations for the transmission of FMD virus within and between wildlife and cattle populations, FMD outbreak incidence was expected to peak during the hot and dry season, the more so since empirical evidence of relatively high incidence of FMD outbreaks during the dry season in cattle and wildlife have been reported in southern Africa [51, 54]. However in the present analysis of primary FMD outbreaks in Zimbabwe FMD incidence peaked during the cold and dry season, a time of year when water and forage are still widely available, and was at its lowest during the hot and dry season, the time of year when water and forage are extremely scarce. Moreover no correlation was detected between cumulated rainfall at the end of a wet season (which would influence subsequent water and forage availability) and the number of primary FMD outbreaks during the subsequent 12 month period. These results imply that water availability is not the only driver of the contacts within and between cattle and wildlife populations that generate FMD outbreaks in cattle.

Another important factor that can influence wildlife/livestock contacts and therefore the spillover of FMD into cattle populations is the cropping calendar, determined by the timing of the rainy season but also dependent on the type of crop planted and local practices. During the rainy and growing crops season, cattle are carefully herded far from the fields in order to avoid crop destruction, an important source of conflicts within local communities. Depending on local contexts (density of cattle and fields, geo-spatial arrangements), cattle can then be taken closer to buffalo population. Later, when crops have been harvested, herders tend to leave herds roaming more freely potentially promoting more buffalo/cattle contacts. Finally, a complementary hypothesis for the occurrence of FMD outbreaks during the cold dry season is that it coincides with the time when buffalo calves become infected by FMD and potentially excrete abundantly the virus [44, 51].

In period of instability (e.g. war of independence, socio-economic collapse) the number of FMD outbreaks increased (Figure 4). It decreased in periods of stability, whether during strong state-control era (Rhodesian time—period 1) when animal health surveillance was a tool to do much more than its initial purpose (i.e. controlling black populations [16] or during the post-independence period (period 3). Globally, the state of veterinary fences surrounding national parks for FMD control and the capacity of veterinary services in Zimbabwe has followed the same patterns: well-maintained (but not 100% proof) and efficient respectively during period of stability, with few FMD outbreaks; and with heavy deterioration to almost complete destruction and few means to implement their activities during period of instability [55–57].

An alternative explanation to the role of the wildlife/livestock interface in triggering FMD outbreaks would be that FMD got endemic in the SEL cattle population and that outbreaks would appear from time to time in this region or less often further away (e.g. in Central regions) through cattle movements. The recent observed increase in FMD outbreaks in southern Africa since 2000 [38] has been linked with a potential increase in silent circulation of FMD strains in cattle. The number of outbreaks observed in the last period (particularly post-2008) could indicate this more complex situation. It has also been partly associated with the economic instability in Zimbabwe since the beginning of the century that has prevented the continuation of an efficient FMD surveillance and control system (e.g. heavily deteriorated state of fences, lack of fence maintenance and mean to implement FMD ring vaccination around detected outbreaks). However, given the present dataset one cannot test this hypothesis. If verified, this second process (i.e. cattle endemicity) would obscure the historical patterns that we observed in our primary outbreak dataset in the years to come and political and economic stability would be necessary to control it. It would mean that the spatial and seasonal distribution of FMD primary outbreaks may also be influenced by cattle populations’ movements and interactions (including transboundary movements).

This study provides arguments to further support the role of wildlife/livestock interfaces in the transmission and spread of FMD using a historical dataset and complement the results of recent FMD molecular studies in the region [38]. It also indicates the variability existing in different types of wildlife/livestock interfaces and the drivers that could explain this variability. It underscores the necessary social and economic stability necessary for animal disease control, and how environmental drivers such as rainfall and therefore climate change can impact disease occurrence, requesting flexible FMD control programs. These hypotheses had already been suggested for Zimbabwe and/or southern Africa but rarely tested against a historical dataset. We suggest that risk-based management of FMD such as vaccination should be concentrated around certain areas (e.g. proximity with National Parks) and implemented before peak period for wildlife/livestock contacts (i.e. during the cold/hot dry season) given the short immunological coverage provided by current FMD vaccines in the region [58]. The management of surface water and grazing could also help managing wildlife/livestock contacts and therefore the risk of FMD spillover. Finally, we call for more participative and inclusive interactions between the various stakeholders involved in livestock production systems as current non-beneficiaries of FMD control carry most of its burden. Control measures should be negotiated with local stakeholders, especially small-scale farmers in order to propose measures acceptable by all and without sidelining anyone.

## Data Availability

The datasets used for rainfall and seasonal analyzes during the current study are available in Agroclimatic database management system, FAOClim-NET (http://geonetwork3.fao.org/climpag/agroclimdben.php). The FMD outbreaks database was obtained from the Department of Livestock and Veterinary Services of the Ministry of Agriculture, Mechanization and Irrigation Development—Zimbabwe (DLVS). Data are available from the corresponding author upon reasonable request and with permission of DLVS.

## Abbreviations

FMD: foot and mouth disease
SEL: South East Lowveld
OIE: World Organisation for Animal Health
FAO: United Nations organization for food and agriculture
PCP: Progressive Control Pathway
SAT: South African Territories
DLVS: Department of Livestock and Veterinary Services
LLR: Logarithm Likelihood Ratio
MLC: most likely cluster
GLM: Generalized Linear Model
